# Efficient production of extracellular pullulanase in *Bacillus subtilis* ATCC6051 using the host strain construction and promoter optimization expression system

**DOI:** 10.1186/s12934-018-1011-y

**Published:** 2018-10-22

**Authors:** Xin Liu, Hai Wang, Bin Wang, Li Pan

**Affiliations:** School of Biology and Biological Engineering, Guangzhou Higher Education Mega Centre, South China University of Technology, Building B6, Panyu District, Guangzhou, 510006 Guangdong People’s Republic of China

**Keywords:** *Bacillus subtilis*, Pullulanase, Protease deficient, Host construction, Promoter optimization

## Abstract

**Background:**

*Bacillus subtilis* has been widely used as a host for heterologous protein expression in food industry. *B. subtilis* ATCC6051 is an alternative expression host for the production of industrial enzymes, and exhibits favorable growth properties compared to *B. subtilis* 168. Extracellular expression of pullulanase from recombinant *B. subtilis* is still limited due to the issues on promoters of *B. subtilis* expression system. This study was undertaken to develop a new, high-level expression system in *B. subtilis* ATCC6051.

**Results:**

To further optimize *B. subtilis* ATCC6051 as a expression host, eight extracellular proteases (*aprE*, *nprE*, *nprB*, *epr*, *mpr*, *bpr*, *vpr* and *wprA*), the sigma factor F (*spoIIAC*) and a surfactin (*srfAC*) were deleted, yielding the mutant *B. subtilis* ATCC6051∆10. ATCC6051∆10 showed rapid growth and produced much more extracellular protein compared to the widetype strain ATCC6051, due to the inactivation of multiple proteases. Using this mutant as the host, eleven plasmids equipped with single promoters were constructed for recombinant expression of pullulanase (PUL) from *Bacillus naganoensis*. The plasmid containing the P_*spovG*_ promoter produced the highest extracellular PUL activity, which achieved 412.9 U/mL. Subsequently, sixteen dual-promoter plasmids were constructed and evaluated using this same method. The plasmid containing the dual promoter P_*amyL*_–P_*spovG*_ produced the maximum extracellular PUL activity (625.5 U/mL) and showed the highest expression level (the dry cell weight of 18.7 g/L).

**Conclusions:**

Taken together, we constructed an effective *B. subtilis* expression system by deleting multiple proteases and screening strong promoters. The dual-promoter P_*amyL*_–P_*spovG*_ system was found to support superior expression of extracellular proteins in *B. subtilis* ATCC6051.

**Electronic supplementary material:**

The online version of this article (10.1186/s12934-018-1011-y) contains supplementary material, which is available to authorized users.

## Background

*Bacillus* species have been widely used for the production of industrial enzymes [1]. *Bacillus* species are regarded as promising host strains with numerous advantages including: non-toxicity, convenience for gene modification and high yields of target proteins, fast growth rate and low nutrient need [2]. Among *Bacillus* species, *Bacillus subtilis* is an attractive expression host due to its desirable features, such as having GRAS (generally recognized as safe) status, naturally efficient secretory system, the background knowledge is available concerning its genetics, physiology and large-scale fermentation processes [3, 4].

Recombinant expression is an important method to facilitate the production of target proteins. Many genetic strategies have been developed to improve the production of recombinant proteins, such as the use of protease-deficient host strains to prevent degradation [5], the deletion of extracellular protein genes to reduce secretion stress [6], and the optimization of promoters and signal peptides [7, 8]. Specifically, *B. subtilis* strains were engineered to serve as extracellular-protease-deficient strains for the overproduction of heterologous proteins such as *B. subtilis* WB600 [9], *B. subtilis* WB700 [10], and *B. subtilis* WB800 [11] (Table 1). These proteases-deficient strains were all constructed from the commonly used model and laboratory strain *B. subtilis* 168 [12]. *B. subtilis* 168 originates from the Marburg strain, which was deposited as *B. subtilis* ATCC 6051 (1930) and *B. subtilis* NCIB 3610 (1951), respectively [13]. Recently, *B. subtilis* ATCC6051 is an alternative expression host for production of industrial enzymes, which exhibits favorable growth properties as compared to the lab strain 168 [14]. Furthermore, gene expression systems of the genus *Bacillus* have been deeply investigated at the genome and transcriptome levels [15, 16]. In addition, the highly-active and controllable promoter is an important regulatory elements in expression systems. Recent research has focused on a novel and effective strategy for the identification of active promoters via screening of chromosomal DNA fragments and using a combinatory approach to construct two or more tandem promoters. Efficient expression vectors have been developed with different promoters like the Pr2 promoter of the sigW gene [17] and the pBL9 promoter of the glvA gene [18], the dual-promoter P_gsiB_–P_HpaII_ system [19] and the P_HpaII_–P_amyQ_′ system [20], etc.

**Table 1:** Protein products from *B. subtilis*

Protein	Yield	Cultivation	Host	References
Alkaline β-mannanase	6041 U/mL	Shake flask	WB600	[21]
l-Asparaginase	407.6 U/mL	3 L bioreactor (fed-batch)	WB600	[22]
β-Glucanase	4840.4 U/mL	Shake flask	WB600	[23]
Alkaline α-amylase	196.35 U/mL	Shake flask	WB600	[24]
Pullulanase	11.7 U/mL	Shake flask	WB600	[25]
α-Acetolactate decarboxylase	135.8 U/mL	5 L fermenter	WB600	[26]
Catalase	8449 U/mL	Shake flask	WB600	[27]
Nattokinase	281 FU/mL	Shake flask	WB600	[28]
Penicillin G acylase	42 U/mL	Shake flask	WB600	[29]
Penicillin G acylase	0.26 U/mL	Shake flask	WB700	[30]
Staphylokinase	337 mg/L	2 L bioreactor (fed–batch)	WB700	[10]
Xylanase	6.93 U/mL	Shake flask	WB700	[31]
Xylanase	8.46 U/mL	Shake flask	WB800	[31]
Pullulanase	26.5 U/mL	Shake flask	WB800	[25]
Nattokinase	292 FU/mL	Shake flask	WB800	[28]
Endoxylanase	55 U/mL	Shake flask	WB800	[32]
α-Amylase	5566 U/mg	Shake flask	WB800	[33]
d-Mannose isomerase (MIase)	51.2 U/mL	3 L fermenter	WB800	[34]

Pullulanase (PUL, EC 3.2.1.41) is a debranching enzyme in the α-amylase family GH13, capable of specifically hydrolyzing a-1,6-glycosidic linkages in pullulan, amylopectin, glycogen, and other related polysaccharides [35]. In food industries, there is a great commercial interest of using pullulanase, especially in the saccharification process together with saccharifying amylases for the production of high-glucose syrup and maltose [36]. Over the years, a large number of pullulanases have been discovered and identified from different microorganisms, and have been successfully heterologously expressed in *Escherichia coli* [37, 38] and various *Bacillus* species [35, 39]. However, only pullulanase derived from few strains such as *Bacillus acidopullulyticus* [40] and *Bacillus naganoensis* has great commercial value; and the production of pullulanase faces many difficulties, such as the expression levels of recombinant pullulanase were yet limit, low yields and low enzyme activity [36]. As shown in Table 1, the protease-deficient *B. subtilis* host strains (WB600, WB800) have been explored for overexpressing *B. naganoensis* pullulanase, but the expression levels are poorer, and cannot satisfy industrial needs. Therefore, the development of an efficient and easy-to-use expression system for the production of PUL is highly desirable.

It has been reported that *B. subtilis* ATCC 6051 could produce large amounts of foam, highly resistant spores, and multiple types of extracellular protease during fermentation [41–44]. In this study, to optimize *B. subtilis* ATCC6051 as an expression host, eight extracellular proteases (*aprE*, *nprE*, *nprB*, *epr*, *mpr*, *bpr*, *vpr* and *wprA*), the sigma factor F (*spoIIAC*) and a surfactin (*srfAC*) were deleted. We found that using this defective strain to express heterologous protein production is superior to the common model laboratory strain. Furthermore, we constructed eleven single-promoter plasmids and sixteen dual-promoter plasmids for PUL expression in *B. subtilis* ATCC 6051∆10. Taken together, we constructed an effective *B. subtilis* expression system by deleting multiple proteases and screening strong promoters, which has high potential for use in industrial applications.

## Results

### Construction of *B. subtilis* ATCC6051 as the heterologous expression host

**Fig. 1:**
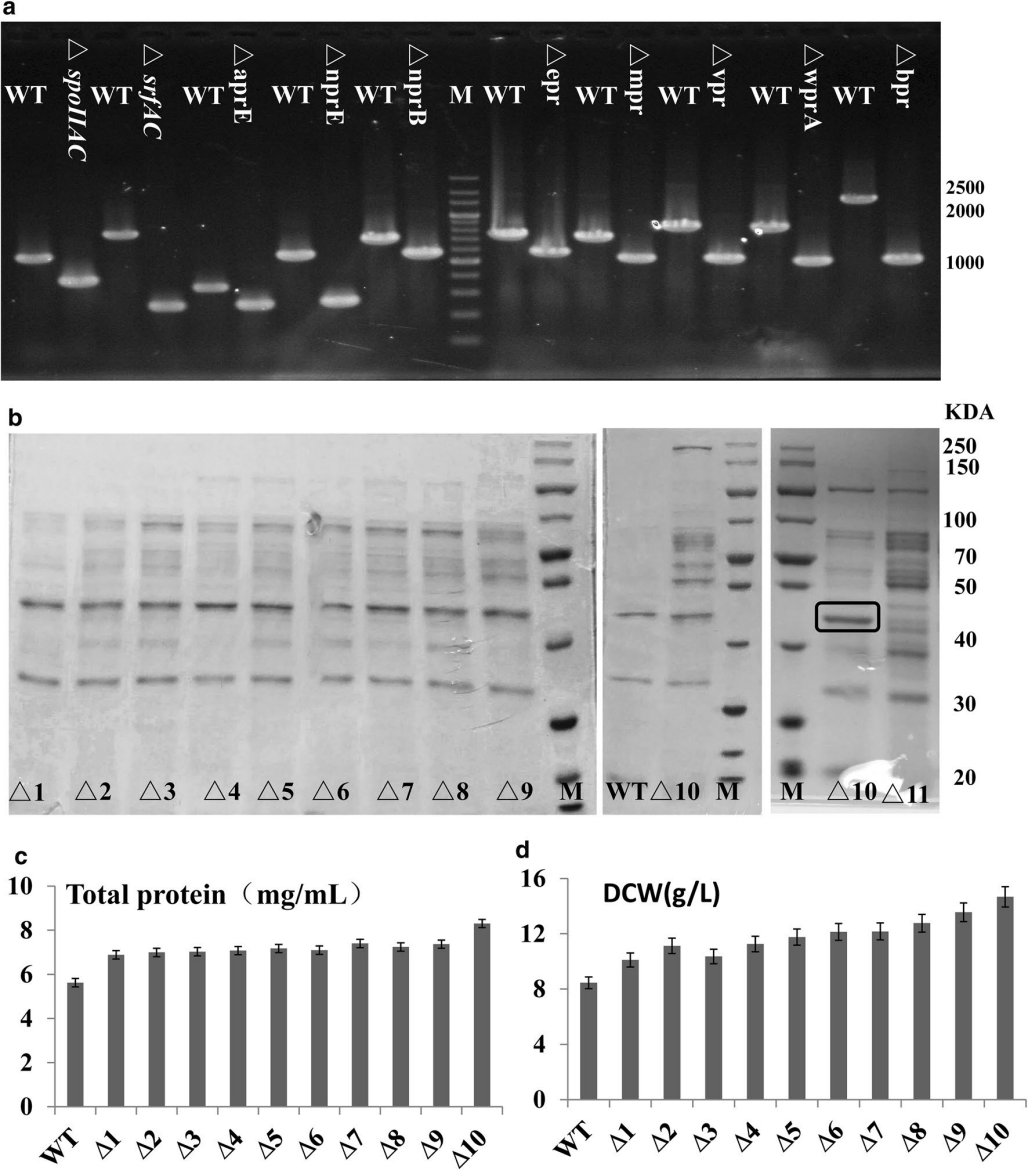
The overview of *B. subtilis* ATCC6051 and the defective strains. **a** PCR verification of the ATCC6051∆10 strain. Lane M, 200 bp DNA ladder. **b** SDS-PAGE analysis of ATCC6051 and eleven defective strains at 48 h. **c** The total protein of ATCC6051 and ten knockout strains at 48 h. **d** The cell growth of ATCC6051 and ten knockout strains at 48 h

In order to develop the wild type strain *B. subtilis* ATCC6051 as the alternative expression host with food-grade safety, we used the temperature-sensitive plasmid to inactivate eight protease genes, one spore-related gene and one surfactin gene, yielding the mutant ATCC6051∆10 (Table 2 and Fig. 1a). Eight extracellular proteases (*aprE*, *nprE*, *nprB*, *epr*, *mpr*, *bpr*, *vpr*, *wprA*) were selected as the targets for deletion, because previous studies have demonstrated that the use of protease-deficient host strains could prevent protein degradation and reduce the secretory stress. As shown in Fig. 1b, the extracellular secreted proteins increased as more genes were deleted. A sigma factor F (*spoIIAC*) and a surfactin (*srfAC*) were also deleted, which affected the production of spores and foam during fermentation, respectively. During the shake flask fermentation process (48 h), *B. subtilis* ATCC6051 produced much foam and required to add 100 µl antifoam (25%, v/v) every 6 h; the mutant ATCC6051∆10 produced much less foam at a controllable level that needed once antifoam at 24 h. After being cultured for 48 h in LB culture medium, the sporulation efficiency of ATCC6051 was 35.33% (122 colonies/300 colonies), while the sporulation efficiency of ATCC6051∆10 was 0% (0 colonies/300 colonies), which shows that ATCC6051∆10 has great resistant to spore formation. Moreover, the mutant ATCC6051∆10 achieved more extracellular protein production compared to that of ATCC6051 (Fig. 1c). And *B. subtilis* ATCC6051∆10 (the dry cell weight of 14.7 g/L) grew faster than the wild-type stain ATCC6051 (Fig. 1d).

**Table 2:** The deficient strains

Strains	Relevant properties	Genes	Gene description	Knockout length (bp)
ATCC6051∆1	∆*spoIIAC*	*spoIIAC*	The gene of RNA polymerase sigma-F factor	371
ATCC6051∆2	∆*spoIIAC*∆*srfAC*	*srfAC*	The gene of surfactin synthase subunit	1024
ATCC6051∆3	∆*spoIIAC*∆*srfAC*∆*aprE*	*aprE*	The gene of serine protease AprE	189
ATCC6051∆4	∆*spoIIAC*∆*srfAC*∆*aprE*∆*nprE*	*nprE*	The gene of bacillolysin	623
ATCC6051∆5	∆*spoIIAC*∆*srfAC*∆*aprE*∆*nprE*∆*nprB*	*nprB*	The gene of neutral protease B	297
ATCC6051∆6	∆*spoIIAC*∆*srfAC*∆*aprE*∆*nprE*∆ *nprB*∆*epr*	*epr*	The gene of minor extracellular protease Epr	326
ATCC6051∆7	∆*spoIIAC*∆*srfAC*∆*aprE*∆*nprE*∆*nprB*∆*epr*∆*mpr*	*mpr*	The gene of glutamyl endopeptidase Mpr	404
ATCC6051∆8	∆*spoIIAC*∆*srfAC*∆*aprE*∆*nprE*∆*nprB*∆*epr*∆*mpr*∆*bpr*	*bpr*	The gene of bacillopeptidase F	1786
ATCC6051∆9	∆*spoIIAC*∆*srfAC*∆*aprE*∆*nprE*∆*nprB*∆*epr*∆*mpr*∆*bpr*∆*vpr*	*vpr*	The gene of extracellular serine protease Vpr	701
ATCC6051∆10	∆*spoIIAC*∆*srfAC*∆*aprE*∆*nprE*∆*nprB*∆*epr*∆*mpr*∆*bpr*∆*vpr*∆*wprA*	*wprA*	The gene of cell wall-associated protease WprA	748
ATCC6051∆11	∆*spoIIAC*∆*srfAC*∆*aprE*∆*nprE*∆*nprB*∆*epr*∆*mpr*∆*bpr*∆*vpr*∆*wprA*∆*hag*	*hag*	The gene of flagellin	698

Additionally, the extracellular proteins increased as more genes were deleted, while some ever-present bands showed that there were still intense in ATCC6051∆10. According to the molecular weights of these bands (Fig. 1b), corresponding proteases were predicted as: superoxide dismutase *sodA* (22.4 kDa), flagellin *hag* (32.6 kDa), RNA degradation presenting factor *YpfD* (42.3 kDa), dihydrolipoyl dehydrogenase *PdhD* (49.8 kDa) and wall-associated protein*WapA* (54.2 kDa). Without affecting the normal growth of the strain, we tried to inactivate a gene corresponding to the most obvious protein bands (*hag*, block mark, Fig. 1b), yielding the mutant ATCC6051∆11: ∆10Δ hag. The extracellular protein became much more obvious, but protein backgrounds of this host strain became much more complicated (Fig. 1b). Therefore, the mutant ATCC6051∆10 may be beneficial for the expression of target proteins, instead of ATCC6051∆11: ∆10Δ*hag*.

### Optimization of promoters for PUL expression in *B. subtilis* ATCC6051∆10

Obtaining highly active promoters is an effective method for increasing the expression of heterologous proteins. The plasmids, which contained the PUL gene from *B. naganoensis* ATCC 53909, eleven promoters (Table 3, Eight promoters were from *B. subtilis*: P43, P_*spovG*_, P_*aprE*_, P_*amyE*_, P_*hag*_, P_*veg*_, P_*nprE*_ and P_*nprB*_; P_*amyL*_ and P_*glvA*_ were from *Bacillus licheniformis*; P_*sigW*_ was from *Bacillus amyloliquefaciens*) and the signal peptide of *amyQ* gene from *B. amyloliquefaciens* (SP_*amyQ*_), were used to construct the secretory expression with the plasmid pBE-MCS in the following: pBEPUL01, pBEPUL02, pBEPUL03, pBEPUL04, pBEPUL05, pBEPUL06, pBEPUL07, pBEPUL08, pBEPUL09, pBEPUL10 and pBEPUL11 (Table 4).

**Table 3:** Properties of promoters used for PUL expression optimization

Promoter	Origin	Properties
P43	*B. subtilis*	Strong constitutive promoter [35]
P_*spovG*_	*B. subtilis*	Promoter of septation protein spovG
P_*aprE*_	*B. subtilis*	Promoter of alkaline protease [19]
P_*amyE*_	*B. subtilis*	Promoter of α-amylase
P_*hag*_	*B. subtilis*	Promoter of flagellin
P_*veg*_	*B. subtilis*	Promoter of protein Veg
P_*nprE*_	*B. subtilis*	Promoter of neutral protease E [19]
P_*nprB*_	*B. subtilis*	Promoter of neutral protease B
P_*amyL*_	*B. licheniformis*	Promoter of α-amylase, strong constitutive promoter [62]
P_*glvA*_	*B. licheniformis*	Promoter ofα-amylase [18]
P_*sigW*_	*B. amyloliquefaciens*	Promoter of RNA polymerase sigma factor SigW [17]

**Table 4:** Strains and plasmids

Plasmid-containing strains	Plasmids	Promoter	Host
PUL0	pBE-MCS	None	*B. subtilis* ATCC6051∆10
PUL1	pBEPUL01	P43	*B. subtilis* ATCC6051∆10
PUL2	pBEPUL02	P_*spovG*_	*B. subtilis* ATCC6051∆10
PUL3	pBEPUL03	P_*aprE*_	*B. subtilis* ATCC6051∆10
PUL4	pBEPUL04	P_*amyE*_	*B. subtilis* ATCC6051∆10
PUL5	pBEPUL05	P_*hag*_	*B. subtilis* ATCC6051∆10
PUL6	pBEPUL06	P_*veg*_	*B. subtilis* ATCC6051∆10
PUL7	pBEPUL07	P_*nprE*_	*B. subtilis* ATCC6051∆10
PUL8	pBEPUL08	P_*nprB*_	*B. subtilis* ATCC6051∆10
PUL9	pBEPUL09	P_*amyL*_	*B. subtilis* ATCC6051∆10
PUL10	pBEPUL10	P_*glvA*_	*B. subtilis* ATCC6051∆10
PUL11	pBEPUL11	P_*sigW*_	*B. subtilis* ATCC6051∆10
PUL201	pBEPUL201	P_*spovG*_–P_*spovG*_	*B. subtilis* ATCC6051∆10
PUL202	pBEPUL202	P_*spovG*_–P43	*B. subtilis* ATCC6051∆10
PUL203	pBEPUL203	P_*spovG*_–P_*amyL*_	*B. subtilis* ATCC6051∆10
PUL204	pBEPUL204	P_*spovG*_–P_*sigW*_	*B. subtilis* ATCC6051∆10
PUL205	pBEPUL205	P_*amyL*_–P_*amyL*_	*B. subtilis* ATCC6051∆10
PUL206	pBEPUL206	P_*amyL*_–P43	*B. subtilis* ATCC6051∆10
PUL207	pBEPUL207	P_*amyL*_–P_*spovG*_	*B. subtilis* ATCC6051∆10
PUL208	pBEPUL208	P_*amyL*_–P_*sigW*_	*B. subtilis* ATCC6051∆10
PUL209	pBEPUL209	P43–P43	*B. subtilis* ATCC6051∆10
PUL210	pBEPUL210	P43–P_*spovG*_	*B. subtilis* ATCC6051∆10
PUL211	pBEPUL211	P43–P_*amyL*_	*B. subtilis* ATCC6051∆10
PUL212	pBEPUL212	P43–P_*sigW*_	*B. subtilis* ATCC6051∆10
PUL213	pBEPUL213	P_*sigW*_–P_*sigW*_	*B. subtilis* ATCC6051∆10
PUL214	pBEPUL214	P_*sigW*_–P43	*B. subtilis* ATCC6051∆10
PUL215	pBEPUL215	P_*sigW*_–P_*spovG*_	*B. subtilis* ATCC6051∆10
PUL216	pBEPUL216	P_*sigW*_–P_*amyL*_	*B. subtilis* ATCC6051∆10

**Fig. 2:**
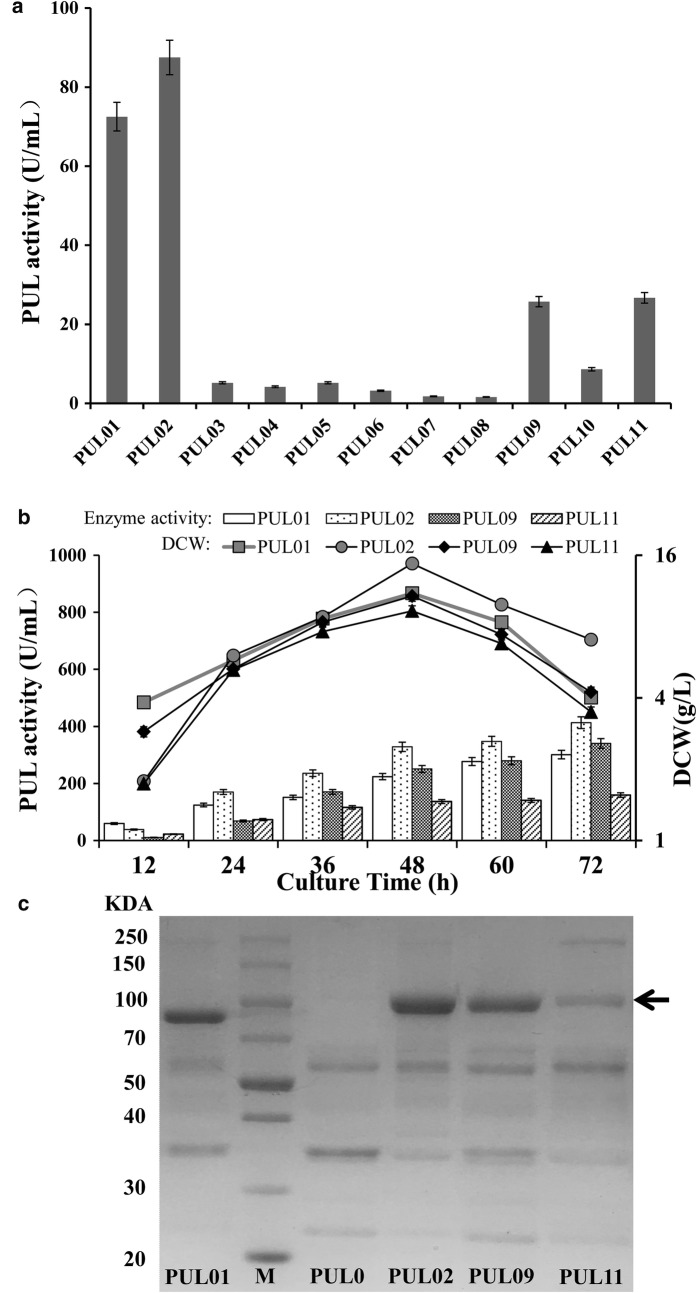
Extracellular PUL expression driven by the single-promoter systems in ATCC6051∆10. **a** PUL activity of eleven single promoters in in well plates. **b** Time profiles of extracellular PUL activity and dry cell weight for the four high viability promoters in ATCC6051∆10. **c** SDS-PAGE analysis of extracellular PUL expression by these single-promoter plasmid-containing strains. The protein bands of PUL were indicated by arrows

The eleven plasmids described above were used to transform *B. subtilis* ATCC6051∆10 in which ten genes were disrupted. The relative strengths of these promoters were determined by measuring the extracellular PUL activities of the eleven plasmid containing strains using orifice plate cultivation. As shown in Fig. 2a, the extracellular PUL activity of strains PUL01 through PUL11 were 72.5, 87.5, 5.2, 4.2, 5.1,3.2, 1.8, 1.6, 25.7, 8.6 and 26.7 U/mL, respectively. Four (PUL01, PUL02, PUL09 and PUL11) of the 11 plasmid containing strains showed higher activity than others, and the PUL02 containing promoter P_*spovG*_ showed the highest PUL activity. Furthermore, the above four strains with high enzyme activity were expanded in shake-flask cultivation, the cell growth and PUL activities of these strains were compared. According to the results (Fig. 2b), the extracellular pullulanase activities were detected in different phases during the expression process, these four strains reached the stationary phase after 48 h. In the late phase of expression process, the maximum PUL activities were reached 412.9, 340.4, 301 and 159.5 U/mL under the control of promoter P_*spovG*_, P_*amyL*_, P43 and P_*sigW*_, respectively. Meanwhile, the activity of PUL expressed by these four plasmid-containing strains indicated that the vitality effect among these four promoters were: P_*spovG*_> P_*amyL*_> P43 > P_*sigW*_. In addition, the dry cell weight of plasmid-containing strain PUL11 (P_*sigW*_) showed the lowest expression level (9.32 g/L) and the plasmid-containing strain PUL02 (P_*spovG*_) showed the highest expression level (14.76 g/L) at 48 h. As shown in Fig. 2c, SDS-PAGE analysis of supernatant proteins was carried out to verify these results, and the thicknesses of the appropriate bands (around 100 kDa) were in agreement with the PUL activity values.

### PUL expression of dual promoters replacement in *B. subtilis* ATCC6051∆10

Having compared eleven single promoters and identified promoters P_*spovG*_, P_*amyL*_, P43 and P_*sigW*_ as superior among them, with P_*spovG*_ being the strongest, dual-promoter plasmids were constructed to further increase PUL expression. To create the dual promoter constructs, promoters P_*spovG*_, P_*amyL*_, P43 and P_*sigW*_ were combined with each other, yielding plasmids pBEPUL201, pBEPUL202, pBEPUL203, pBEPUL204, pBEPUL205, pBEPUL206, pBEPUL207, pBEPUL208, pBEPUL209, pBEPUL210, pBEPUL211, pBEPUL212, pBEPUL213, pBEPUL214, pBEPUL215 and pBEPUL216, respectively (Table 4). The sixteen dual-promoter plasmids were used to transform *B. subtilis* ATCC6051∆10, yielding the sixteen corresponding plasmid-containing strains PUL201 through PUL216.

**Fig. 3:**
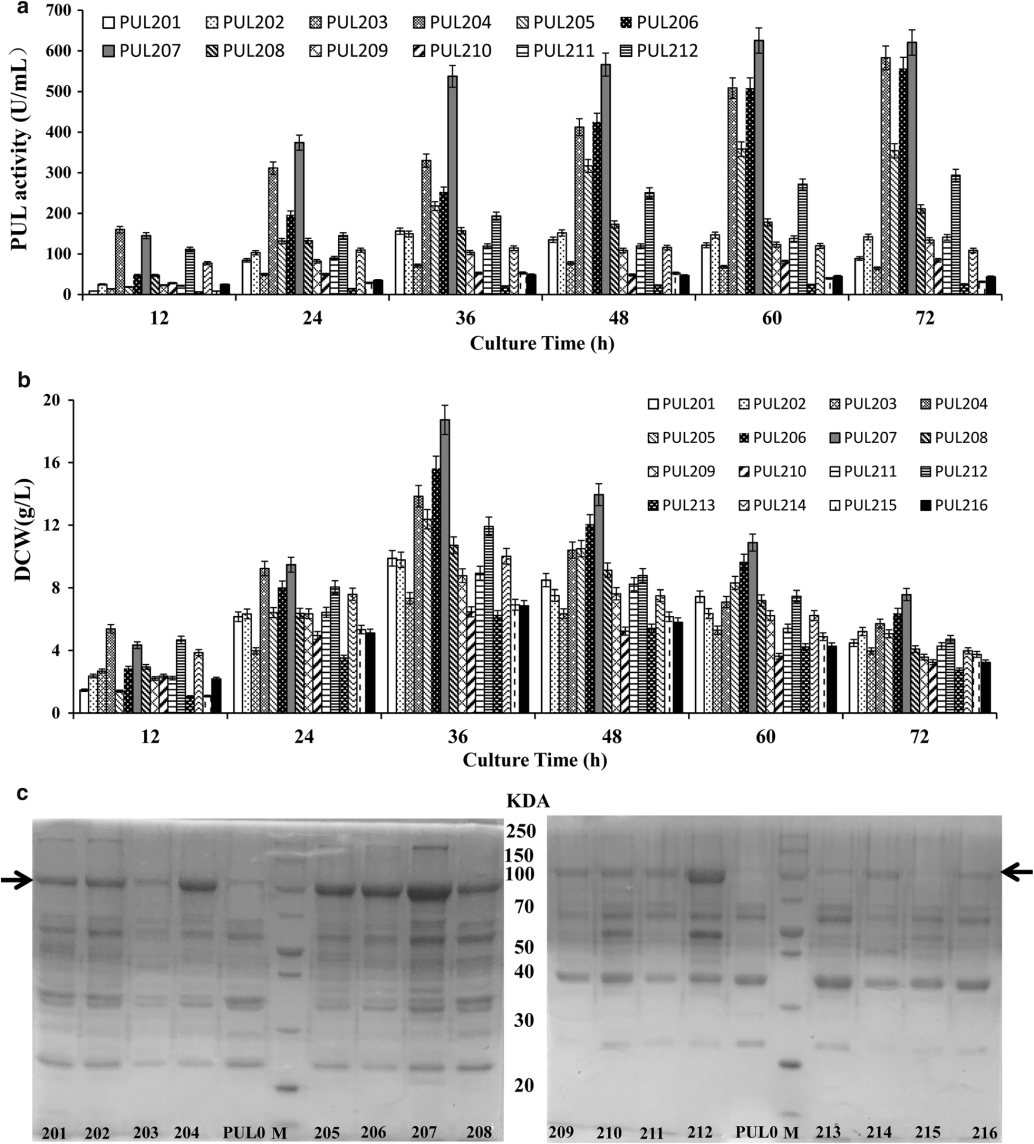
Extracellular PUL expression driven by the dual-promoter systems in ATCC6051∆10. **a** Time profiles of extracellular PUL activity for the sixteen dual-promoters in ATCC6051∆10. **b** Time profiles of the dry cell weight for the sixteen dual-promoters in ATCC6051∆10. **c** SDS-PAGE analysis of extracellular PUL expression by these dual-promoter plasmid-containing strains. The protein bands of PUL were indicated by arrows

The expression strengths of the sixteen dual promoters were measured using shake-flask experiments similar to those used to assess the single promoters. As shown in Fig. 3a, the extracellular maximum PUL activities of plasmid-containing strains PUL201 through PUL216 during the expression process were 156.2 (P_*spovG*_–P_*spovG*_), 151.7 (P_*spovG*_–P43), 77.1 (P_*spovG*_–P_*amyL*_), 582.9 (P_*spovG*_–P_*sigW*_), 358.1 (P_*amyL*_–P_*amyL*_), 556.2 (P_*amyL*_–P43), 625.5 (P_*amyL*_–P_*spovG*_), 210.8 (P_*amyL*_–P_*sigW*_), 133.6 (P43–P43), 84.5 (P43–P_*spovG*_), 141 (P43–P_*amyL*_), 293.5 (P43–P_*sigW*_), 25.44 (P_*sigW*_–P_*sigW*_), 119.7 (P_*sigW*_–P43), 53 (P_*sigW*_–P_*spovG*_) and 48.3 (P_*sigW*_–P_*amyL*_) U/mL, respectively. Obviously, the plasmid-containing strain PUL207, which harbored the plasmid pBEPUL207 with the dual promoter P_*amyL*_–P_*spovG*_, showed the highest PUL activity. This activity was almost 1.49-fold and 1.84 the activity produced by plasmid-containing strain PUL02 and PUL09, which expressed PUL using the P_*amyL*_ and P_*spovG*_ promoter, respectively. Two more plasmid-containing strains PUL204 (P_*spovG*_–P_*sigW*_) and PUL206 (P_*amyL*_–P43) also have higher enzyme activity than the single promoter. Moreover, The dry cell weights of the sixteen plasmid-containing strains peaked at 36 h, and decreased substantially from 48 to 72 h. In particular, the plasmid-containing strain PUL207 (18.73 g/L) was much higher than that of the other fifteen plasmid-containing strains (Fig. 3b). SDS-PAGE analysis of the supernatant of the culture broth also indicated a distinctive protein band at around 100 kDa for the positive recombinant but not the negative control (Fig. 3c), consistent with the results of activity assay.

## Discussion

*Bacillus subtilis* species are usually used as the host strains for heterologous protein expression. However, the host strains can produce many different types of extracellular protease which might degrade the target protein [1], and many protease-deficient strains display favorable heterologous protein production [43–45]. The multiple proteases-deficient host strains have been constructed, such as *B. subtilis* WB600, WB700 and WB800 [5, 46]. And the deficiency of proteases genes (*nprE*, *nprB*, *mpr*, *vpr*, *epr*, *bpr*, *wprA*, *aprE*) in *B. subtilis* improved the expression of target proteins obviously. Furthermore, Sigma F, encoded by *SpoIIAC* gene, controls the formation of forespore [47]. Gutierrez et al. showed that inactivation of sigma F could largely block the sporulation of *B. subtilis* [48]. Moreover, *B. subtilis* produces surfactin, an amphiphilic molecule which could accumulate at gas–liquid interfaces and induce foam during fermentation [49, 50]. *B. subtilis* ATCC 6051 produces a large amount of foam during fermentation, which has an extreme effect on fermentation process control and may lead to contamination [51]. This shows that surfactin may be a major mediator for foam formation, which may affect the protein secretion. In this study, eight extracellular proteases genes (*nprE*, *nprB*, *mpr*, *vpr*, *epr*, *bpr*, *wprA* and *aprE*), the sigma factor F gene (*SpoIIAC*) and the surfactin gene (*srfAC*) were knocked out to obtain the mutant ATCC 6051∆10.

Due to the great demand of industrial applications, the overexpression of pullulanase has received extensive attention in recent years [52]. Among the effective solutions and prerequisites are the selection of new bacterial strains or the improvement of bacterial strains [53] and *B. subtilis* strains are well known for their ability to secrete a large number of useful extracellular enzymes [54]. In previous reports, several protease-deficient *B. subtilis* host strains have been explored for overexpressing *B. naganoensis* pullulanase, including WB600 [28], WB800 [22], and *B. subtilis* CCTCC M 2016536 (*srfC*, *spoIIAC*, *nprE*, *aprE* and *amyE* genes deletion) [20]. But these hosts showed poor expression of pullulanase and the extracellular activities reached 90.7 U/mL, which was the highest ever reported [20]. Secretory expression of PUL was achieved in ten-genes deficient *B. subtilis* ATCC 6051∆10, and deletion of these ten genes indeed enhanced the production of PUL. This study provided a novel *B. subtilis* host, which has the potential for the heterologous expression of different proteins.

Obviously, promoter as an important regulatory element directly influences the expression efficiency of heterologous protein and strong promoters are usually used to achieve gene high-level expression [20]. In recent years, some previous reports have shown that many promoters in *B. subtilis* have been used for the expression of *B. naganoensis* pullulanase. As known, Wang et al. [55] used three widely promoters (P43, P_*apr*_ and P_*amy*_) to select the best promoter for PUL expression in *B. subtilis*, and the result indicated that P_*apr*_ (2.82 U/mL) is the most suitable for the production of PUL. Song et al. [35] constructed plasmids under the promoter P_*HpaII*_ and P43, resulting in the extracellular PUL activity of 24.5 U/mL (P43). Other studies have shown that two or more tandem promoters can significantly increase the level of heterologous protein expression. Zhang et al. [20] showed that the tandem promoter P_*HpaII*_–P_*amyQ′*_ (90.7 U/mL) were found to elevate PUL productivity by 1.49 fold compared to the single P_*amyQ′*_ (60.9 U/mL) system. Although the above promoters are used as strong promoters in *B. subtilis*, these promoters resulted in various levels of expression of the PUL reporter protein. In our work, a set of *B. subtilis* recombinants involving different combination of various promoters were constructed for heterologous expression of PUL from *B. naganoensis*. Compared with the previous reports, the promoters P_*spovG*_ (412.9 U/mL), P_*amyL*_ (340.4 U/mL), and P43 (301 U/mL) can significantly increase the activity of PUL. Moreover, the dual promoters P_*amyL*_–P_*spovG*_ (625.5 U/mL), P_*spovG*_–P_*sigW*_ (582.9 U/mL) and P_*amyL*_–P43 (556.2 U/mL) showed the higher extracellular PUL activity than the single promoters. Thus, the above promoter expression systems may have the potential for the production of useful proteins in *B. subtilis*.

## Conclusion

In this study, the ten-genes-deficient strain *B. subtilis* ATCC6051∆10 was obtained by overlap gene knockout. Compared with *B. subtilis* ATCC 6051, the mutant ATCC6051∆10 had less foam during fermentation under the same conditions, displayed greater resistant to spore formation, achieved 1.48 times more extracellular protein production and 1.73 times more dry cell weight. Then, eleven single-promoter plasmids and sixteen dual-promoter plasmids were constructed and evaluated using shake-flask cultivation. By host strain construction and promoters optimization, the maximum PUL activity of 625.5 U/mL was achieved under the control of the dual promoter P_*amyL*_–P_*spovG*_. This study provides a valuable expression system with the potential application for industrial production of PUL, as well as expression of other proteins.

## Materials and methods

### Strains, plasmids and primers

The detailed information for genes, strains and plasmids were shown in Additional file 1: Table S1, and primers sequences were listed in Additional file 1: Table S2. The following concentrations of antibiotics were used for selection: 100 μg/mL ampicillin (Amp), 5 μg/mL erythromycin (Erm) and 20 μg/mL kanamycin (Kan). Luria–Bertani (LB) medium consisted of 1% tryptone, 0.5% yeast extract, and 1% NaCl, with the pH 7. The flask fermentation medium contained LB medium with 1% corn starch.

### Gene prediction

All the annotated gene sequences of the proteases from *B. subtilis* were selected from the NCBI database (https://www.ncbi.nlm.nih.gov/), and were used to find the corresponding genes in *B. subtilis* ATCC6051 by Blast tool (http://blast.ncbi.nlm.nih.gov).

### Construction of the knockout vectors

The knockout vectors were constructed by a previously reported method [56], using the *mpr* gene as an example (pKS2-*mpr*, Additional file 1: Figure S1**)**. The upstream and downstream homologous arms (about 500 bp) of *mpr* gene, named *mpr* (L) and *mpr* (R), were amplified using the primers of F-mpr-1/R-mpr-1 and F-mpr-2/R-mpr-2 (Additional file 1: Table S2) respectively, and the amplified fragments were further purified and recovered. By splicing overlap extension PCR method using the primers of F-mpr-1/R-mpr-2, the *mpr* (L) and *mpr* (R) were fused to form the fragment *mpr* (L + R), which was then cloned into the pKS2 (5094 bp) at the *EcoR* V restriction site. Other knockout vectors were constructed following the same method.

### Gene knockout

The competent cells of *B. subtillis* were prepared by the method reported [57]. The competent cells were electro-transformed at 2.5 kV with 10 μL knockout plasmid solution (100 ng/μL), maintained in 900 μL recovery medium (LB medium added with 1% sorbitol) at 30 °C and 100 rpm for 4–6 h. The cells were spread onto LB plate with 5 μg/mL erythromycin, incubated at 30 °C for 16–24 h, and the transformants were selected by PCR and plasmid extraction verification. The positive transformants were inoculated into LB liquid medium containing 5 μg/mL erythromycin, cultured at 37 °C for 12 h and plated onto erythromycin LB solid medium, which was further incubated at 37 °C to screen the single-crossover colonies. After PCR verification, the positive clones were subcultured in LB liquid medium without erythromycin at 30 °C for 12–16 h. At last, the cultures were incubated in LB plates at 37 °C for 20 h, and each single colony was inoculated onto a LB plate with erythromycin, as well as one without erythromycin. If a colony grew on the LB plate without erythromycin and was inhibited on LB plate with erythromycin, this strain could be the double crossover one, which would be further verified by PCR.

### Construction of the PUL expression vector

The PUL expression vector was constructed based on the *pul* gene from *B. naganoensis* ATCC 53909, the signal peptide of *amyQ* gene from *B. amyloliquefaciens* (SP_*amyQ*_), the P43 promoter and the terminator of *amyE* gene (T_*amyE*_) from *B. subtillis* 168. First, the fragments of P43, SP_*amyQ*_, *pul* and T_*amyE*_ were amplified by the primers (Additional file 1: Table S2) and purified, respectively. Second, the purified fragments of P43, SP_*amyQ*_, *pul* gene and were fused by PCR to obtain the P43SPT fragment. Finally, the P43SPT fragment was inserted into the pBE-MCS plasmid at *Eco*RI and *Xba*I sites, which was then transformed into *Escherichia coli* HST08 to obtain the expression plasmid pBEPPUL01 (Additional file 1: Figure S2). Using the In-Fusion HD Cloning Plus kit [58], plasmid fragment one was joined with promoter fragments P_*spovG*_, P_*aprE*_, P_*amyE*_, P_*hag*_, P_*veg*_, P_*nprE*_, P_*nprB*_, P_*amyL*_, P_*glvA*_, P_*sigW*_ and sixteen dual-promoter fragments to yield plasmids pBEPUL2 to pBEPUL11 and pBEPUL201 to pBEPUL216, respectively.

### Analysis methods

According to a previously described method [59], the activity of PUL was assayed by the concentration of reducing sugars liberated into the reaction mixture and one unit of pullulanase was defined as the amount of enzyme required to produce the reducing sugar equivalent to 1 L/mol glucose per min. Data represent the average of three independent experiments, and error bars indicate the standard error of the mean value.

The cell growth was evaluated by measuring the dry cell weight (DCW) [60]. DNA and the proteins were determined by agarose gel electrophoresis and sodium dodecyl sulfate–polyacrylamide gel electrophoresis (SDS-PAGE) [61], respectively.

## Additional file

**Additional file 1: Figure S1.** Construction procedure of the knockout vector (pKS2-mpr). All knockout vectors were constructed as shown. **Figure S2.** Construction procedure of the promoter plasmid (pBEPUL01). All expression plasmids were constructed as shown. **Table S1.** Genes, bacterial strains and plasmids used in this study. **Table S2.** primers used in this study.

## Abbreviations

LB: Luria–Bertani
Amp: ampicillin
Kan: kanamycin
Erm: erythromycin
PCR: polymerase chain reaction
PUL: pullulanase
*spollAC*: the gene of RNA polymerase sigma-F factor
*srfAC*: the gene of surfactin synthase subunit
*aprE*: the gene of subtilisin E
*nprE*: the gene of bacillolysin
*nprB*: the gene of neutral protease B
*epr*: the gene of minor extracellular protease Epr
*mpr*: the gene of glutamyl endopeptidase Mpr
*vpr*: the gene of extracellular serine protease Vpr
*bpr*: the gene of bacillopeptidase F
*wprA*: the gene of cell wall-associated protease WprA
*hag*: the gene of flagellin
amyE: the gene of α-amylase
amyQ: the gene of α-amylase from *B. amyloliquefaciens*
SDS-PAGE: sodium dodecyl sulfate-polyacrylamide gel electrophoresis
