# Supplementary material for: Efficient production of extracellular pullulanase in Bacillus subtilis ATCC6051 using the host strain construction and promoter optimization expression system
Source: Microb Cell Fact. 2018 Oct 22;17:163. doi: 10.1186/s12934-018-1011-y (PMC6196424; doi:10.1186/s12934-018-1011-y)
Supplement: Supplementary file 1 — Additional file 1: Figure S1. Construction procedure of the knockout vector (pKS2-mpr). All knockout vectors were constructed as shown. Figure S2. Construction procedure of the promoter plasmid (pBEPUL01). All expression plasmids were constructed as shown. Table S1. Genes, bacterial strains and plasmids used in this study. Table S2. primers used in this study. [file 12934_2018_1011_MOESM1_ESM.docx]

**Additional file for**

**Efficient production of extracellular pullulanase in *Bacillus subtilis* ATCC6051 using the host strain construction and promoter optimization expression system**

Xin Liu1, Hai Wang1, Bin Wang3, Li Pan*

E-mail: l.x64@mail.scut.edu.cn (Xin Liu), 201520133692@mail.scut.edu.cn (Hai Wang), btbinwang@scut.edu.cn (Bin Wang), btlipan@scut.edu.cn (Li Pan)

School of Biology and Biological Engineering, South China University of Technology, Guangzhou, 510006, Guangdong, China

co-first authors: Xin Liu and Hai Wang

*Corresponding authors: Li Pan

E-mail: btlipan@scut.edu.cn

Address: Building B6, School of Biology and Biological Engineering, South China University of Technology, Guangzhou Higher Education Mega Centre, Panyu District, Guangzhou City, 510006, Guangdong Province, P. R. China.

Tel.: +86-20-39380616

**Additional figures**

**
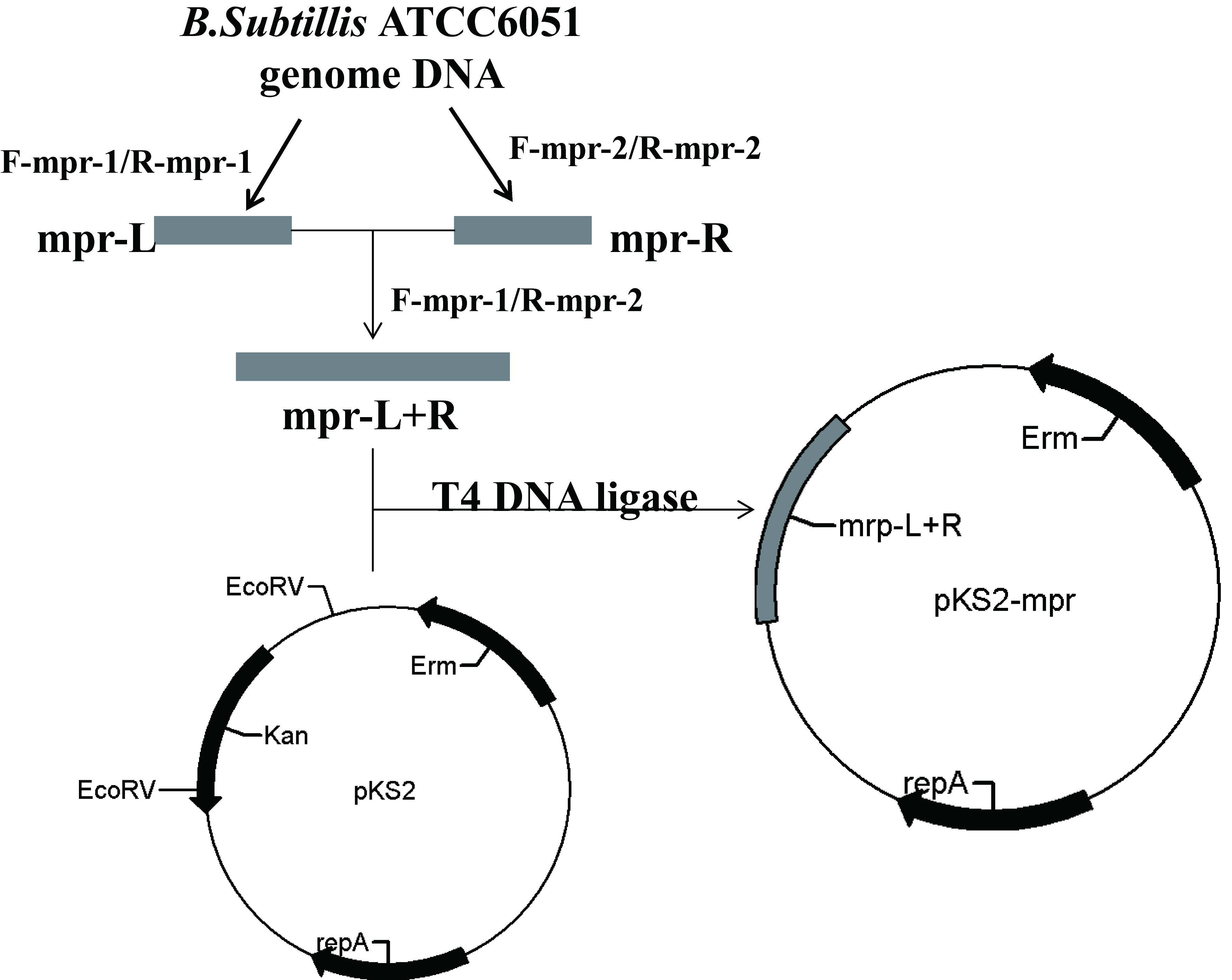
**

**Figure S1. Construction procedure of the knockout vector (pKS2-mpr).** All knockout vectors were constructed as shown.

**
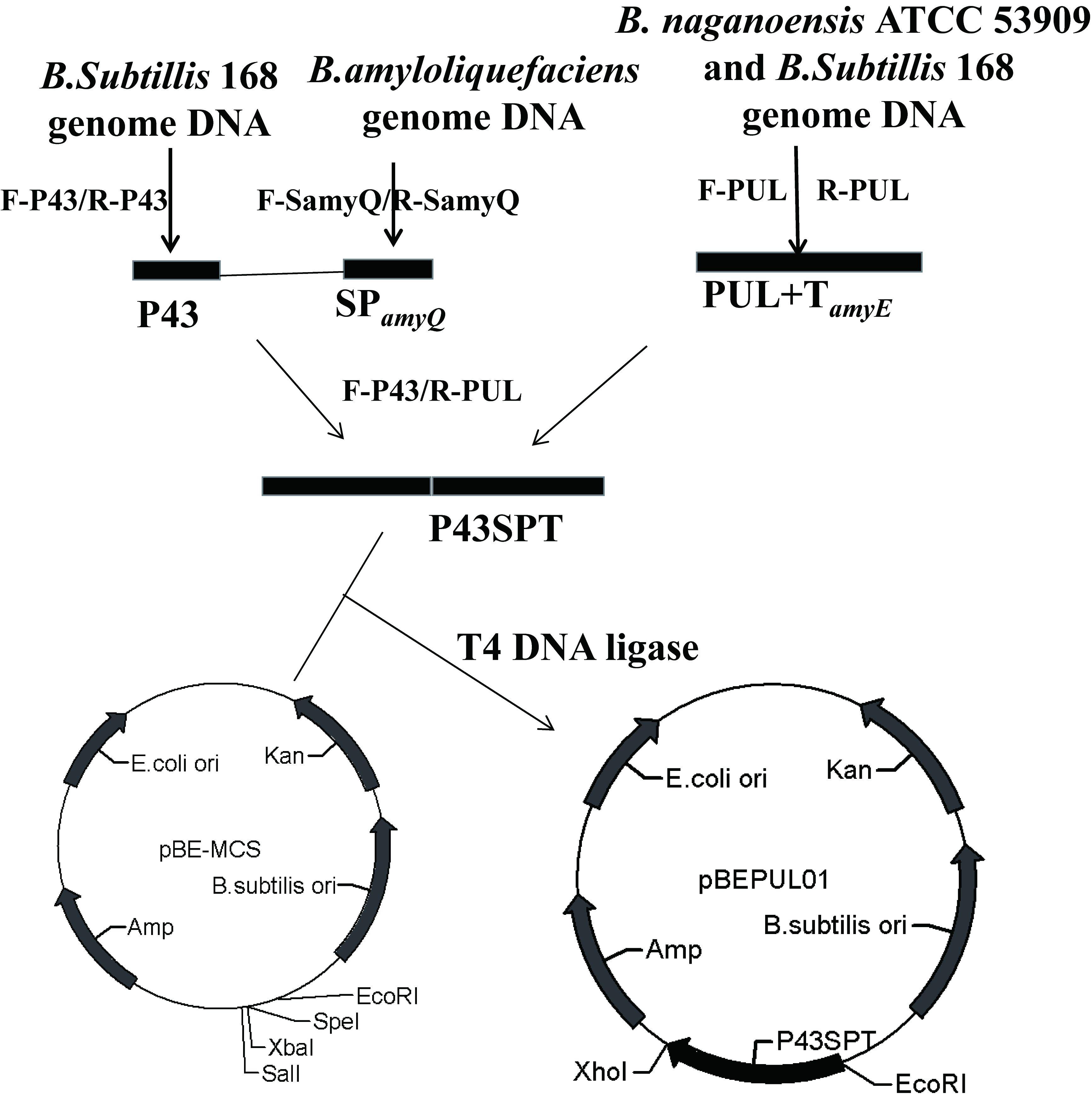
**

**Fig. S2 Construction procedure of the promoter plasmid (pBEPUL01).** All expression plasmids were constructed as shown.

**Additional tables**

**Table S1 Genes, bacterial strains and plasmids used in this study**

| **Genes** | **Characteristics** | **Source** |
| --- | --- | --- |
| *amyE* | The gene of α-amylase; |  |
| *SpoVG* | The gene of septation protein; |  |
| *veg* | The gene of protein Veg; |  |
| *amyL* | The gene of α-amylase from *Bacillus licheniformis*; |  |
| *glvA* | The gene of 6-phospho-alpha-glucosidase from *Bacillus licheniformis*; |  |
| *amyQ* | The gene of α-amylase from *Bacillus amyloliquefaciens*; |  |
| *sigW* | The gene of RNA polymerase sigma factor SigW from *Bacillus amyloliquefaciens*; |  |
| **Strains** |  |  |
| *E. coli* HST08 | competent cells; dam^-^, dcm^-^ | Takara |
| *Bacillus subtilis* 168 | wild type | Our laboratory |
| *Bacillus subtilis* ATCC6051 | wild type | NBRC  (Biological Resource Center) |
| *Bacillus naganoensis ATCC 53909* | wild type | NBRC |
| *Bacillus subtilis* ATCC6051∆1 | *∆spollAC* | This work |
| *Bacillus subtilis* ATCC6051∆2 | *∆spollAC*、∆*srfAC* | This work |
| *Bacillus subtilis* ATCC6051∆3 | *∆spollAC*、∆*srfAC*、∆*aprE* | This work |
| *Bacillus subtilis* ATCC6051∆4 | *∆spollAC*、∆*srfAC*、∆*aprE*、∆*nprE* | This work |
| *Bacillus subtilis* ATCC6051∆5 | *∆spollAC*、∆*srfAC*、∆*aprE*、∆*nprE*、∆*nprB*、 | This work |
| *Bacillus subtilis* ATCC6051∆6 | *∆spollAC*、∆*srfAC*、∆*aprE*、∆*nprE*、∆*nprB*、∆*epr* | This work |
| *Bacillus subtilis* ATCC6051∆7 | *∆spollAC*、∆*srfAC*、∆*aprE*、∆*nprE*、∆*nprB*、∆*epr*、∆*mpr* | This work |
| *Bacillus subtilis* ATCC6051∆8 | *∆spollAC*、∆*srfAC*、∆*aprE*、∆*nprE*、∆*nprB*、∆*epr*、∆*mpr*、∆*bpr* | This work |
| *Bacillus subtilis* ATCC6051∆9 | *∆spollAC*、∆*srfAC*、∆*aprE*、∆*nprE*、∆*nprB*、∆*epr*、∆*mpr*、∆*bpr*、∆*vpr* | This work |
| *Bacillus subtilis* ATCC6051∆10 | *∆spollAC*、∆*srfAC*、∆*aprE*、∆*nprE*、∆*nprB*、∆*epr*、∆*mpr*、∆*bpr*、∆*vpr*、∆*wprA* | This work |
| *Bacillus subtilis* ATCC6051∆11 | *∆spollAC*、∆*srfAC*、∆*aprE*、∆*nprE*、∆*nprB*、∆*epr*、∆*mpr*、∆*bpr*、∆*vpr*、∆*wprA*、∆*hag* | This work |
| **Plasmids** |  |  |
| pKS2 | *E. coli* and *B. subtillis* shuttle vector; Kan^r^ ,Erm^r^ | Our laboratory |
| pKS2- *spollAC* | pKS2*- spollAC* (L + R); to knock out *spollAC* | This work |
| pKS2- *srfAC* | pKS2*- srfAC* (L + R); to knock out *srfAC* | This work |
| pKS2- *aprE* | pKS2*- aprE* (L + R); to knock out *aprE* | This work |
| pKS2- *nprE* | pKS2*- nprE* (L + R); to knock out *nprE* | This work |
| pKS2- *nprB* | pKS2*- nprB* (L + R); to knock out *nprB* | This work |
| pKS2- *epr* | pKS2*- epr* (L + R); to knock out *epr* | This work |
| pKS2- *mpr* | pKS2*- mpr* (L + R); to knock out *mpr* | This work |
| pKS2- *bpr* | pKS2*- bpr* (L + R); to knock out *bpr* | This work |
| pKS2- *vpr* | pKS2*- vpr* (L + R); to knock out *vpr* | This work |
| pKS2- *wprA* | pKS2*- wprA* (L + R); to knock out *wprA* | This work |
| pKS2- *hag* | pKS2*- hag* (L + R); to knock out *hag* | This work |
| pBE-MCS | *E. coli* and *B. subtillis* shuttle vector; Kan^r^,Amp^r^ | Our laboratory |

**Table S2 primers used in this study**

| **Primers** | **Sequences(5’→3’)** | **Restriction Sites** |
| --- | --- | --- |
| F-spollAC-1 | TACGGTTTGAACATCAATTG |  |
| R- spollAC -1 | GTCTGTCGTAAGATCCGATTACCCTGCTTG |  |
| F- spollAC -2 | AATCGGATCTTACGACAGACCAAACAAGAC |  |
| R- spollAC -2 | CAGCAGGCACTCCTGACACT |  |
| F-srfAC-1 | AACAAGCATCACTGCGTTAC |  |
| R- srfAC -1 | CAGTCGGCCCCCATATCAAAGCCGTTTTCA |  |
| F- srfAC -2 | TTTGATATGGGGGCCGACTGAGGGAACAGT |  |
| R- srfAC -2 | TTGTGCGATTGTTTCTTCCA |  |
| F-aprE-1 | CGTTTGTCCAAGTCGGGTGC |  |
| R-aprE-1 | GCATTTCTCACGCTCTTAATAACTCAATCG |  |
| F-aprE-2 | ATTAAGAGCGTGAGAAATGCCATAAGGAAC |  |
| R-aprE-2 | GTGAGAAGCAAAAAATTGTG |  |
| F-nprE-1 | TCAACTTTAGCGGCATCAGT |  |
| R-nprE-1 | AGACGCTGTTTGGCGTCACCCAAGAAACAG |  |
| F-nprE-2 | GGTGACGCCAAACAGCGTCTGGTGATTTGC |  |
| R-nprE-2 | TCAGGCTGCTGAAGGTCATC |  |
| F-nprB-1 | AAAAGTCACCGCAGAAGCC |  |
| R-nprB-1 | TTGTTTGCCGTCACCTGACACGCCGATT |  |
| F-nprB-2 | tgtcaggtgaCGGCAAACAATGGAACAAT |  |
| R-nprB-2 | CGAGCCTTCACCGTATAAAT |  |
| F-epr-1 | AAGGCCGGAAAGGAAACC |  |
| R-epr-1 | GACGTTTGTCCGTCGTGCCAAGGCTCATA |  |
| F-epr-2 | tggcacgacgGACAAACGTCCAGCTTCGC |  |
| R-epr-2 | CGTCTTCAAATTGGTGCTCTT |  |
| F-mpr-1 | TCTTTGGAAAGCCGATAACA |  |
| R-mpr-1 | GTATCAGACCGTGCTGGAGATTCTGGTGC |  |
| F-mpr-2 | tctccagcacGGTCTGATACAAAGCCGAT |  |
| R-mpr-2 | AACGAGATGTGATGGGTTAC |  |
| F-bpr-1 | TATGAGGATGGTTGGGACG |  |
| R-bpr-1 | GATTGGGCTGCCGCCGTGATGCTTGGTT |  |
| F-bpr-2 | atcacggcggCAGCCCAATCACGGTCAC |  |
| R-bpr-2 | CATCTCGGACGATCACTTCT |  |
| F-vpr-1 | AAAGGCATCAAGGTGGCG |  |
| R-vpr-1 | ACGAGCGAATGCTGAAGAGTAGGAGCCGA |  |
| F-vpr-2 | actcttcagcATTCGCTCGTCTCACCTGG |  |
| R-vpr-2 | GCTTGACTTGCCTTTGTTCG |  |
| F-wprA-1 | TTCACAGCGGAGGATGGA |  |
| R-wprA-1 | GCGGAATCAATGACGGGACTGATTGTAAAG |  |
| F-wprA-2 | agtcccgtcaTTGATTCCGCAGGTTCCG |  |
| R-wprA-2 | GACATTCAGCCGCCCGTA |  |
| F-hag-1 | ATTTCGCGGATCACTTCAT |  |
| R-hag-1 | CGCAGCAGGTCAACATTCTTTCTCAGGCTTC |  |
| F-hag-2 | AAGAATGTTGACCTGCTGCGTCATCTCCC |  |
| R-hag-2 | TGCGAATTTATTGGCTCCC |  |
| F-P43 | CGGAATTCGGTTTACTTATTTTTTTGCC | *EcoR*Ⅰ |
| R-P43 | CCACTAGTGTGTACATTCCTCTCTTACCTATAA | *Spe*I |
| F-SamyQ | GAATGTCGACATGATTCAAAAACGAAAGCG |  |
| R-SamyQ | TGTCCATCTCGAGGGCTGATGTTTTTGTAATCG |  |
| F-PUL | CCCTCGAGGATGGGAACACCACAAACATCGT | *Xho*I |
| R-PUL | GCTCTAGACTAATGATGATGATGATGATGTTTACCATCAGATGGGCTTACTTC | *Xba*Ⅰ |
| PspovG-F | CGGAATTCACGGACAATATTTTGACACTC | *EcoR*Ⅰ |
| PspovG-R | GGACTAGTAGTAGTTCACCACCTTTTCCC | *Spe*I |
| PaprE-F | CGGAATTCACGGAAATAGCGAGAGATGATA | *EcoR*Ⅰ |
| PaprE-R | GGACTAGTACGCGTCCCTCTCCTTTTGC | *Spe*I |
| PamyE-F | CGGAATTCTGATTGTGAAGCTGGCTTAC | *EcoR*Ⅰ |
| PamyE-R | GGACTAGTTCTTGACACTCCTTATTTGAT | *Spe*I |
| Phag-F | CGGAATTCGGATTTTTTTATTTTTGTATTAAC | *EcoR*Ⅰ |
| Phag-R | GGACTAGTTGTTTTGTTCCTCCCTGAATA | *Spe*I |
| Pveg-F | CGGAATTCCTCCGTAATACGCTGACAAG | *EcoR*Ⅰ |
| Pveg-R | GGACTAGTTGCATCCACCTCACTACATT | *Spe*I |
| PnprE-F | CGGAATTCCCAACACGAACAACAATCCT | *EcoR*Ⅰ |
| PnprE-R | GGACTAGTAATAAATCCCCCTTTTTGAAAA | *Spe*I |
| PnprB-F | CGGAATTCGATCGGCTGGCCAGAATAG | *EcoR*Ⅰ |
| PnprB-R | GGACTAGTAACACCACATCCTTCCTATTT | *Spe*I |
| PamyL-F | CGGAATTCGCAACGTTCGCAGATGCTGC | *EcoR*Ⅰ |
| PamyL-R | GGACTAGTACATATGATATTGTATAAATATTCC | *Spe*I |
| PglvA-F | CGGAATTCAGCCCTCCGGCCAACCCGT | *EcoR*Ⅰ |
| PglvA-R | GGACTAGTAAGCCCCCTTATAAGCGTTTAC | *Spe*I |
| PsigW-F | CGGAATTCTTGGGCTATAGCCAAGCGGT | *EcoR*Ⅰ |
| PsigW-R | GGACTAGTGTTTTATCTTACCTCTGCCCTT | *Spe*I |

The restriction sites are underlined.
